# Broadband and wide-angle RCS reduction using a 2-bit coding ultrathin metasurface at terahertz frequencies

**DOI:** 10.1038/srep39252

**Published:** 2016-12-16

**Authors:** Lanju Liang, Minggui Wei, Xin Yan, Dequan Wei, Dachuan Liang, Jiaguang Han, Xin Ding, GaoYa Zhang, Jianquan Yao

**Affiliations:** 1 School of Opto-Electronic Engineering, Zaozhuang University, Zaozhuang 277160, China; 2 College of Precision Instrument and Opto-electronics Engineering, Tianjin University, Tianjin 300072, China; 3 Center for Terahertz Waves, College of Precision Instrument and Opto-electronics Engineering, Tianjin University, Tianjin 300072, China; 4 School of Information Science and Engineering, University of Jinan, Jinan 250022, China

## Abstract

A novel broadband and wide-angle 2-bit coding metasurface for radar cross section (RCS) reduction is proposed and characterized at terahertz (THz) frequencies. The ultrathin metasurface is composed of four digital elements based on a metallic double cross line structure. The reflection phase difference of neighboring elements is approximately 90° over a broadband THz frequency. The mechanism of RCS reduction is achieved by optimizing the coding element sequences, which redirects the electromagnetic energies to all directions in broad frequencies. An RCS reduction of less than −10 dB bandwidth from 0.7 THz to 1.3 THz is achieved in the experimental and numerical simulations. The simulation results also show that broadband RCS reduction can be achieved at an incident angle below 60° for TE and TM polarizations under flat and curve coding metasurfaces. These results open a new approach to flexibly control THz waves and may offer widespread applications for novel THz devices.

Compared to microwave and infrared frequencies, THz radar has some advantages, such as high spatial resolution, greater secrecy, strong penetrating capability, extremely ultra-wide bandwidth, among others^1^,^2^,^3^,^4^. Therefore, THz radar has great prospects in remote sensing and military application. RCS reduction has received increasing attention in recent years with the rapid development of detection and stealth technology. The metamaterial absorber, which can offer nearly perfect absorption, has been successfully demonstrated as a good candidate at THz frequencies^5^,^6^,^7^,^8^,^9^,^10^,^11^,^12^. In 2011, Iwaszczuk *et al*. proposed a flexible absorber with distinct capabilities to achieve RCS reduction at normal incidence^13^. However, this structure exhibits a narrow -band and sensitivity to the angle of incidence, which obviously limits its practical applications. In addition, the absorber uses the loss components of the effective to absorb the energy of incident waves, which transforms electromagnetic energy into heat. However, the change of the object temperature will inevitably increase its possible the detection by infrared detectors.

Several designs for special structures have been proposed to realize broadband RCS reduction at microwave frequencies^14^,^15^,^16^,^17^,^18^,^19^,^20^,^21^. Edalati *et al*. designed and tested a non-absorptive, aperiodic frequency selective surface to achieve wideband and wide-angle RCS reduction^22^. Zheng *et al*. designed and characterized a broadband chessboard structure to reduce the RCS^23^. Li *et al*. proposed to achieve a wideband RCS reduction using a two-dimensional phase gradient metasurface^24^. To improve the bandwidth of RCS reduction and obtain large freedom to design a structure, Cui *et al*. proposed the coding metasurface, where in the RCS reduction was below −10 dB over a broad frequency range from 7.5 GHz to 15 GHz^25^. This special metasurface may significantly simplify the design of specific device functionalities^26^,^27^,^28^,^29^,^30^,^31^. However, RCS reduction at different incident angles has not been reported using coding metasurface. The above broadband RCS reduction structures have been characterized at microwave frequencies. Therefore, designing a new route to reduce RCS at broadband frequencies and wide -angles, remains an important topic and a key issue at THz frequencies.

In this paper, we propose a novel method that uses 2-bit ultrathin coding metasurface to reduce RCS over broadband THz frequencies and wide incident angles for TE and TM polarizations. The reflected energy of the coding metasurface was scattered in various directions by optimizing the coding of the sequences, which significantly reduced the RCS. The characteristics of broadband RCS reduction were measured by a THz time-domain radar system and simulated by the CST Microwave Studio. Its performance at different incident angles was numerically simulated. The proposed metasurface also singnificantly reduced the RCS significantly in an ultra-wide frequency for oblique incidences. In contrast to previous approaches, our metasurface for RCS reduction has the advantages of easy fabrication, broad bandwidths, and wide-angles.

## Results

### Theoretical analysis

The mechanism to reduce the RCS is achieved by redirecting reflective the EM energies in all directions through the digital elements of the coding metasurface^25^.

**Figure 1:**
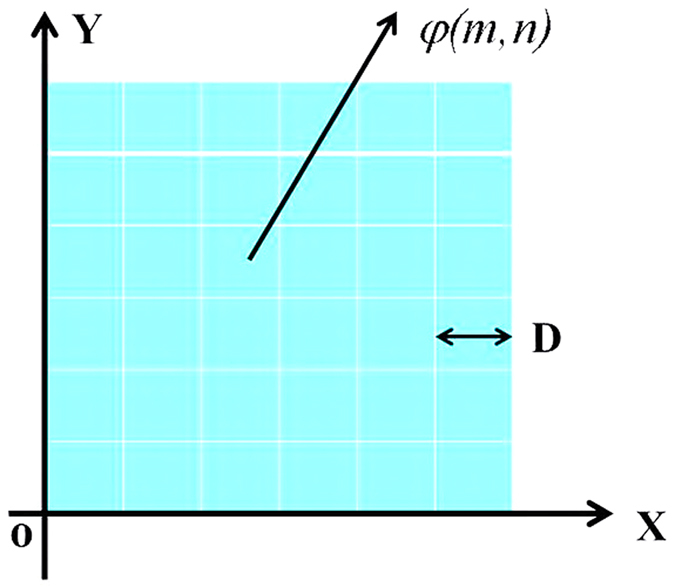
Diagram of the 2 bit coding metasurface, which contains N × N equal-size lattices with dimension D, each lattice is occupied by one of the “00”, “01”, “10” and “11” digital elements.

A diagram of the 2 bit coding metasurface is shown in Fig. 1. The metasurface is composed of *N* × *N* equal-sized lattices with dimension *D*. Each lattice is occupied by one of the “00”, “01”, “10” and “11” digital elements. *φ(m, n*) is the scattering phase of the lattice, which is 0, *π*/2, *π*, and 3*π*/2.

Under the normal incidence of plane waves, the far-field pattern function of the coding metasurface can be expressed as


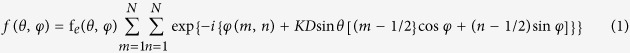


where *θ* and *φ* are the elevation and azimuth angles of an arbitrary direction, respectively, and *f*_*e*_(*θ, φ*) is the pattern function of a lattice. The directivity function *Dir(θ, φ*) of the metasurface is


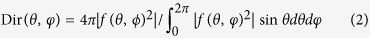


where the *f*_*e*_(*θ, φ*) term is eliminated because of the phase difference between the “00” and “10”, or “01” and “11” digital elements. By treating each digital element as a dipole radiation source, the far-field radiation characteristic can be explained by the interference and superposition principle of electromagnetic wave for these digital elements. In the design process, the reflection responses of these digital elements have basically identical amplitudes, but the reflection phase different is approximately 180° between “00” and “10” or “01” and “11” in a wideband frequency. Hence, the *f*_*e*_(*θ, φ*) term has been eliminated because of the destructive interference between these digital elements. Therefore, the best RCS reduction can be achieved by optimizing the coding sequences of the “00”, “01”, “10”, and “11” lattices.

### Design and simulated of the coding metasurface

We optimize the sequences of the “00”, “01”, “10” and “11” elements with particle-swarm optimization algorithm to reduce the RCS by redirecting the reflective EM waves to all directions using the four types of digital elements.

**Figure 2:**
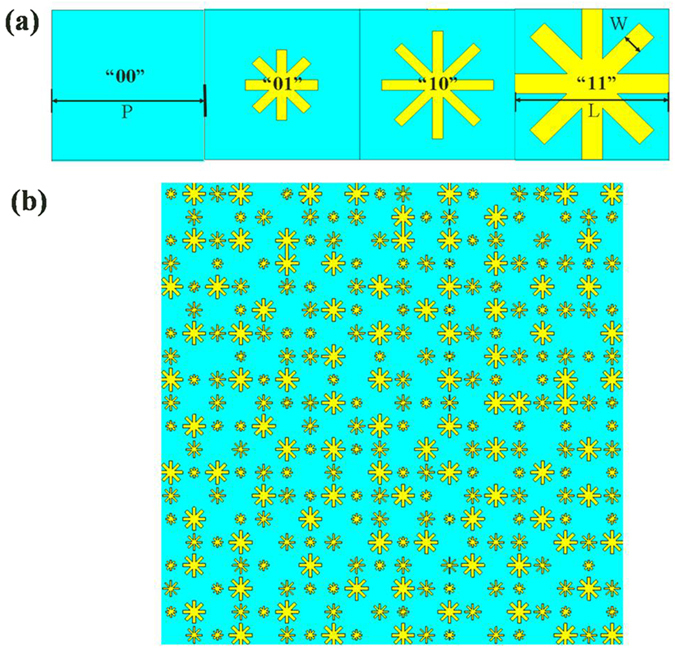
(**a**) Four basic “00”, “01”, “10”, and “11” digital elements with different metallic structure sizes. For the “01” element, *w* = 8 μm, *L* = 56 μm, for the “10” element, *w* = 8 μm, *L* = 86 μm, for the “11” element, *w* = 16 μm, *L* = 120 μm, and *p* = 120 μm, and for the “00” element, there is no metallic structure. (**b**) Schematic of the proposed 2 bit coding metasurface.

We evaluate the characteristics of the proposed metasurface by simulating the far-field scattering and reflection phase through full wave simulation using CST Microwave Studio. The proposed digital element is depicted in Fig. 2(a). The structure, which is composed of double cross lines metallic film, is patterned on a polyimide (PI) film (thickness of *h* = 40 μm) with a dielectric constant of 3.1 and a loss tangent of 0.05. The bottom layer is a metallic film with a thickness of 200 nm. The proposed coding metasurface is composed of “00”, “01”, “10”, and “11” digital elements, and these elements with different metallic structure sizes, as shown in Fig. 2(b). For the “01” digital element, *w = *8 μm, and *L* = 56 μm, for the “10” digital element, *w = *8 μm, *L* = 86 μm; for the “11” digital element, *w = *16 μm, *L* = 120 μm; and the period of the metallic structure is *p* = 120 μm; and the “00” element does not contain the metallic structure.

**Figure 3:**
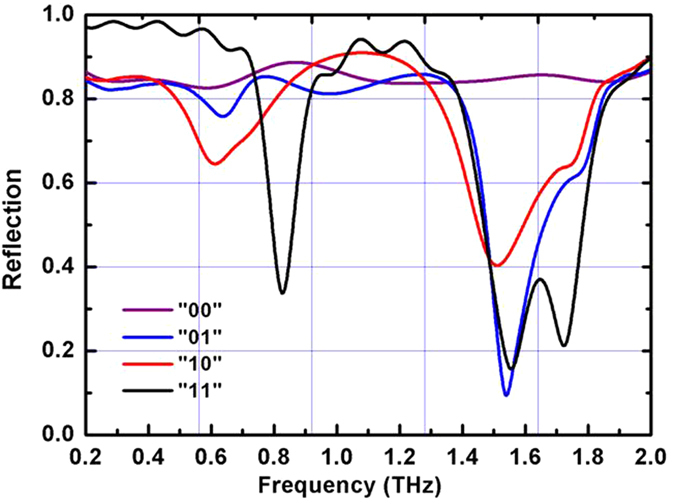
Reflection spectra of the 2-bit coding metasurface for different digital elements at normal incident angle.

**Figure 4:**
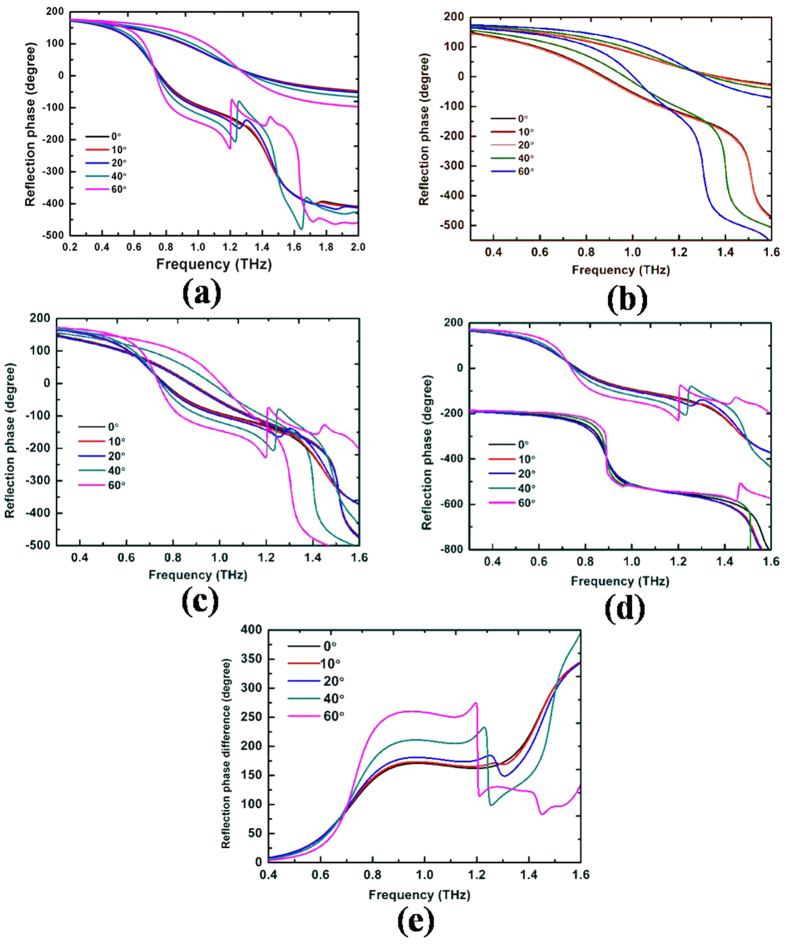
Simulated reflection-phase of digital elements at different incident angles. (**a**) “00” and “10” digital elements. (**b**) “00” and “01” digital elements. (**c**) “01” and “10” digital elements. (**d**) “10” and “11” digital elements. (**e**) Simulated reflection-phase difference of “00” and “10” elements digital elements at different incident angles.

The basic digital elements are “00”, “01”, “10” and “11”, whose reflection responses have almost identical amplitudes, but the reflection phase difference of neighboring digital elements is approximately 90° in a wide THz band from 0.8 THz to 1.4 THz, as shown in Figs 3 and 4. Hence, the *f*_*e*_(*θ, φ*) term has been eliminated with destructive interference.

The reflection phase difference of neighboring digital elements is approximately 90° in a wide THz band from 0.7 THz to 1.5 THz by tailoring the geometrical dimensions of the metallic double cross line structure, as shown in Fig. 4. The RCS is reduced by redirecting the electromagnetic wave to all directions using the four types of digital elements. We optimized the sequences of the “00”, “01”, “10”, and “11” elements using the particle-swarm optimization algorithm. The proposed 2 bit coding metasurface combination of 20 × 20 double cross metallic line unit cells is shown in Fig. 2(b), and the size of a unit cell of the metasurface is 2.4 mm × 2.4 mm.

The reflection phases and their difference of the digital elements were simulated at different incident angles as shown in Fig. 4(a–d). Figure 4 shows that the change in phase difference of each digital element decreases at incidence angles less than 40°, but it is larger than 60° if the incidence angle increases to 60°, particularly for the “10” digital elements. These results will affect the broadband RCS reduction characteristics with increasing incidence angles.

To discuss the effect of the change in phase difference, we calculated the reflection phase difference of the “00” and “10” digital elements at different incident angles, as shown in Fig. 4(e). Figure 4(e) shows that a reflection phase difference of almost 180° is maintained in a wide band of frequencies from 0.76–1.40 THz (to within ±30°) at incidence angles less than 40°. When the incidence angle is increased to 60°, the bandwidth for the difference of almost 180° becomes narrow (only 0.7–0.8 THz and 1.2–1.3 THz). Therefore, the effect of reflection cancellation and diffuse reflection will be reduced, and the backward RCS of the metallic plate cannot be reduced at wideband frequencies for coating the designed coding metasurface.

**Figure 5:**
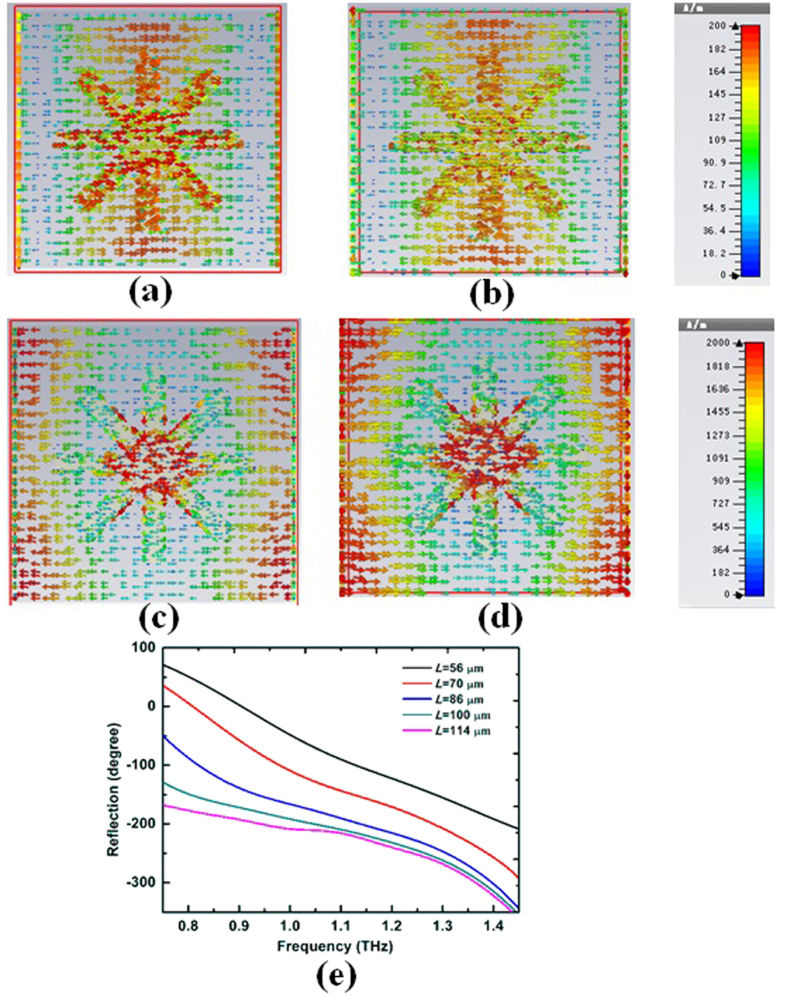
Simulated distribution of the surface current on (**a**) the front metal structure at 0.61 THz. (**b**) The back metal film at 0.61 THz. (**c**) The front metal structure at 1.51 THz. (**d**) The back metal film at 1.51 THz. (**e**) Reflection phase with different lengths *L* and fixed dimensions *w* = 8 μm, *p* = 120 μm for double metallic structure.

Compared to the traditional single-layer structures, the double metallic cross line structure has the advantage of wide-band, wide-angle reflection phase manipulation. The distribution of surface currents for the “10” digital element was simulated as shown in Fig. 5. Figure 5 shows that the surface currents are antiparallel on the front metal structure and back metal film at the resonance frequencies of 0.61 THz and 1.51 THz. The antiparallel currents cause the electromagnetic resonance of the metal structure, therefore the double metallic cross line structure has the advantage of wide-angle reflection phase manipulation. The reflection phase responses of double metallic cross line structure with different lengths *L* are shown in Fig. 5(e). Figure 5(e) shows that the phase curves are almost parallel to the change in *L* at a broadband frequency of 0.75 to 1.45 THz, which is important to guarantee the working bandwidth of the coding unite cells.

Finally, this symmetric arrangement of the unite cells can provide similar phase responses for both TE and TM polarization wave incidences.

**Figure 6:**
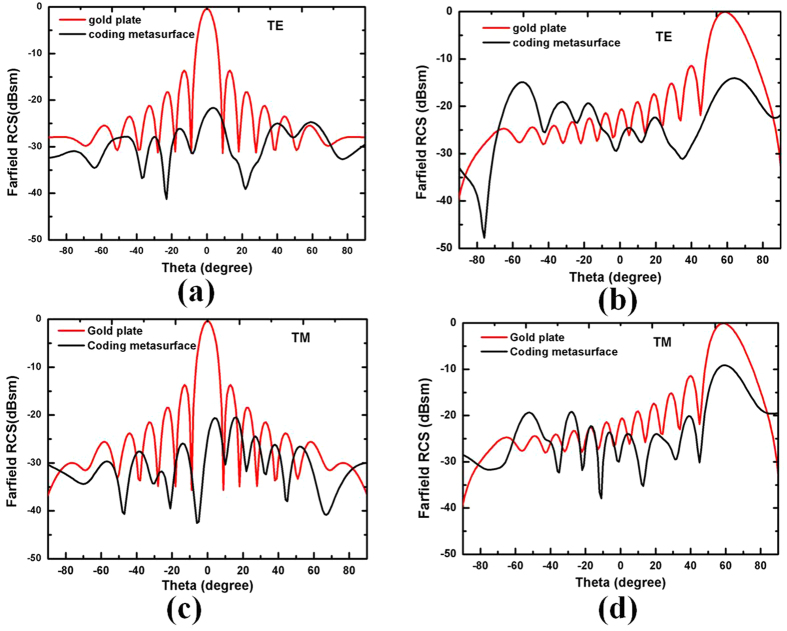
Simulated far-field bi-static RCS distribution for a bare metallic plate and the coding metasurface with TE and TM polarizations at 0.8 THz for (**a**,**c**) normal incidence and (**b**,**d**) incidence angle at −60°.

We determined the performance of the 2 bit coding metasurface by simulating the bi-static RCS distributions of a bare metallic plate as well as a metallic plate coated with a coding metasurface under TE and TM polarizations at 0.8 THz for normal and −60° incidence angles, as shown in Fig. 6(a–d). The metallic plate clearly has a strong backscatter for both polarizations. When the metallic plate is covered with the proposed coding metasurface, diffusion waves are produced for strong reflected energy, and RCS reduction is obtained for both TE and TM polarization. The maximum RCS reduction is more than −22 dB at the normal incidence, and the RCS reduction remains at more than −10 dB at the −60° incidence angle.

**Figure 7:**
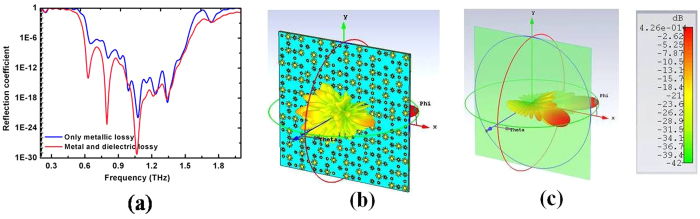
(**a**) Simulated reflection coefficient of the coding metasurface in different situations for only metallic lossy, metal and dielectric lossy respectively. (**b**,**c**) Simulations results of the far-field 3D RCS patterns at 0.8 THz for the incidence angle of −60 degree under TE polarization. (**b**) Coding metasurface (**c**) metallic plate.

Figure 6(a) and (c) show that the metallic plate has larger bi-static RCS than the coding metasurface for the entire reflection angle range from −90° to 90° because the RCS is reduced, redirecting the reflective electromagnetic wave to all directions. For the metallic plate, the reflective electromagnetic wave (EM) energies are equal to the incident EM energies. However, the total reflective EM energies for the coding metasurface are less than the incident EM energies. This result can be explained from Fig. 7. We simulated the reflection coefficient under the metal and dielectric lossy, and only metal lossy of the coding metasurface. The reflection coefficient increases under only metal lossy for the coding metasurface. Thus, the dielectric lossy of the coding metasurface partially absorbs the part incident EM energies. Therefore, the metallic plate has larger bi-static RCS than the coding metasurface in the entire reflection angle range from −90° to 90°.

The far-field 3D RCS patterns were simulated with the coding metasurface and metallic plate at 0.8 THz for the incidence angle of −60 degrees under TE polarization, as shown in Fig. 7(b,c). Figure 7(b,c) show that the metallic plate has a strong backscatter for the angle at 60 degrees, but the scattering energy is notably less for other directions. When the metallic plate is covered with the coding metasurface, diffusion waves are produced for strong reflected energy, and the scattering energy in each direction is approximately equal to that at the angle of 60 degrees. Therefore, the coding metasurface has a larger RCS than the metallic plate in the incidence direction.

**Figure 8:**
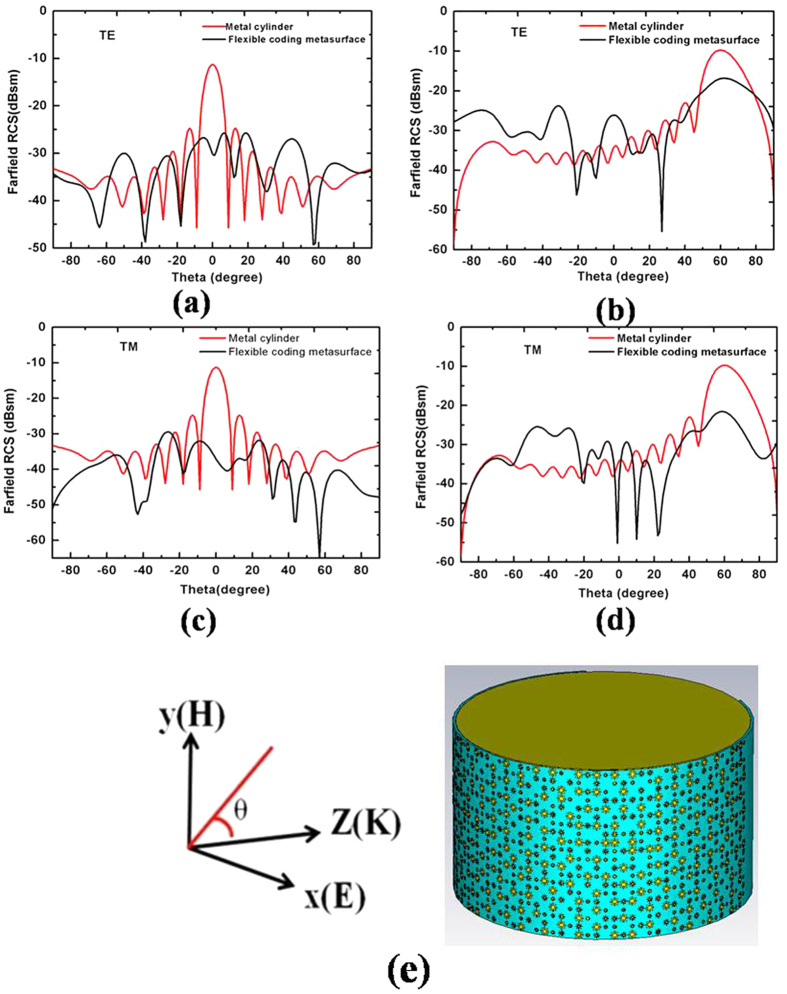
Simulated far-field RCS distribution for PEC cylinder with and without flexible wrapping by the coding metasurface under TE and TM polarizations at 0.8 THz for (**a**,**c**) normal incidence and (**b**,**d**) incidence angle at −60°. (**e**) Schematic of the PEC cylinder with the flexible 2-bit coding metasurface.

The RCS distributions were also simulated under TE and TM polarizations at 0.8 THz for normal and −60° incidence angles, as shown in Fig. 8(a–d). The curve coding metasurface can significantly reduce RCS for both normal and oblique incidences, which is similar to those of the flat coding metasurface. The maximum RCS reduction is more than −25 dB at the normal incidence, and the RCS reduction is closed to −10 dB at the −60 degree incidence angle.

The metallic plate has a strong backscatter for both polarizations. When the metallic plate and PEC cylinder were covered with the proposed flexible coding metasurface, diffusion waves were produced for the strongly reflected energy, and RCS reduction was obtained for TE and TM polarizations.

**Figure 9:**
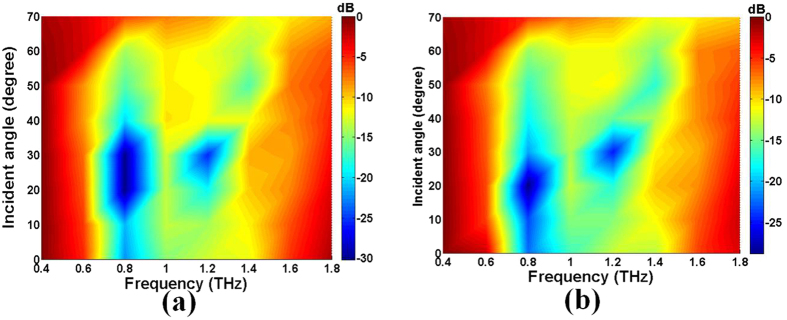
Maps of the simulated RCS reduction in a wide frequency range at different incidence angles for (**a**) TE and (**b**) TM polarizations.

The broadband and wide-angle features of 2 bit coding metasurface are confirmed by simulation to quantify the RCS reduction of the sample using CST at various incidence angles and wide frequencies under TE and TM polarizations, as shown in Fig. 9. An RCS reduction less than −10 dB was achieved in a wide frequency range from 0.7 THz to 1.4 THz for both polarizations, and the RCS reduction was more than −10 dB at the 60 degree incidence. When the incident angle continued to increase, the RCS reduction began to decrease, and the RCS reduction band became narrow. These simulation results validate that the coding metasurface can achieve RCS reduction at wide angles of incidence and a broadband frequencies for both TE and TM waves, the RCS reduction is similar when the incident angle increases from 0 to 60 degrees.

**Figure 10:**
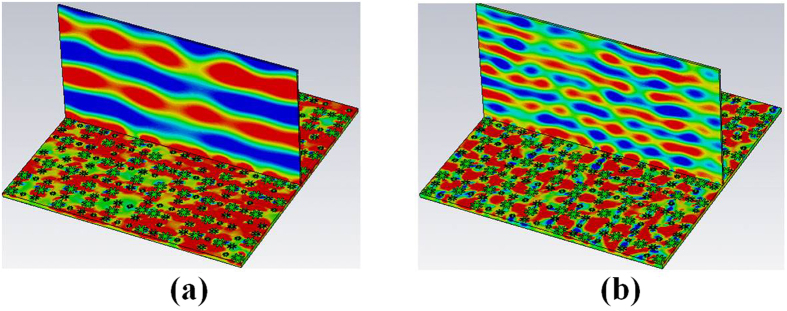
Near-field distribution on a vertical plane to the metasurface at different frequencies of (**a**) 0.6 THz (**b**) 0.8 THz.

We simulated the near-field electromagnetic distribution on a vertical plane to the 2-bit coding metasurface at 0.6 THz and 0.8 THz, as shown in Fig. 10. At 0.6 THz, the reflection-phase difference between the “00” and “10” elements, and “01” and “11” elements is apart from 180 degrees. The effect of cancellation for the reflected wave is found to be insignificant in the normal direction; the former wave is close to a plane wave, so the far-field scattering pattern will produce a single beam, and the RCS reduction will not produce at this frequency. However, at 0.8 THz, the reflection-phase differences of the “00” and “10” elements, “01” and “11” elements are approximately 180 degrees, and the reflected wave is cancelled in the normal direction. Consequently, its wave front is wavy, and the far-field scattering pattern produces two symmetrically oriented directions.

## Experimental results

To further verify the designed structure, a sample of the 2 bit coding metasurface is fabricated through spin-coated, evaporated, lift-off, and peel-off techniques, and the size of the entire sample is 2 inches.

RCS can be expressed as^32^:


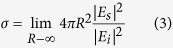



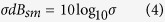


where *E*_*i*_ is the incident field, *E*_*s*_ is the scattering field, and R is the detecting distance.

**Figure 11:**
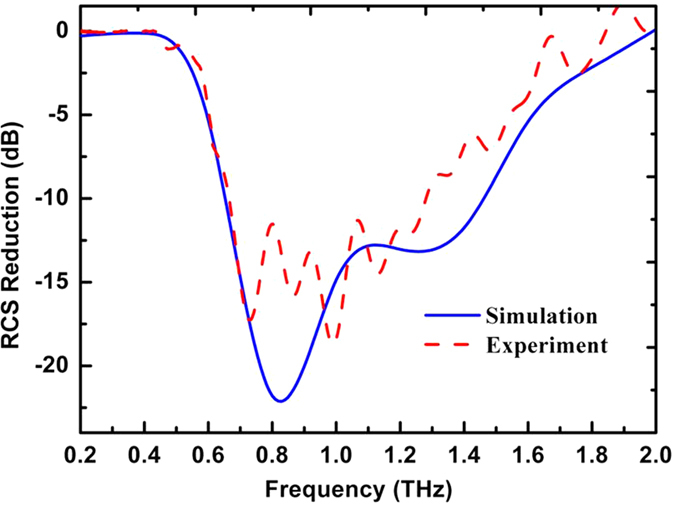
Measured results of RCS reduction over a wide frequency range from 0.2 THz to 1.8 THz.

We simulated the RCS results in a wide frequency range under normal incidence, as shown in Fig. 11(b). The 10 dB bandwidth of the RCS reduction in the backward direction ranges was 0.65 THz to 1.4 THz, which is consistent with that for the phase difference.

The RCS characteristics of the sample are measured through a broad-band time domain THz RCS system^32^,^33^,^34^,^35^. The full width of the beam is 92 mm, the distance between the incident parallel wave and the target object is 1670 mm, and the distance between the scattered THz wave and the detector is 1410 mm. The angle between the incident and scattered waves is 9°. Therefore, our RCS measurements system is bistatic in principle, and reasonably close to a monostatic radar configuration.

The scattering coefficient of the coding metamaterial, metall ball and bare circle metallic plate were measured using broad-band time domain THz RCS system. The diameter of the metal ball is 2.5 cm, and the size of the bare circle metallic plate and designed metasurface sample is 2.2 cm.

According to Equation (3) and (4), we calculate the RCS of the circle metallic plate with and without the coding metasurface. We also calculated the RCS reduction over a wide frequency range from 0.2 THz to 2.0 THz, as shown in Fig. 11(b). The RCS can clearly be reduced by more than 10 dB from 0.7 GHz to 1.4 THz, the maximum of the RCS reduction is 18 dB at 1.0 THz, and the experiment result is consistent with the simulation results. Both simulated and measured results demonstrate that the coding metasurface can effectively reduce the RCS from the bare metal plate in an ultra-wide band at a wide-angle.

## Conclusions

The design of a 2 bit coding metasurface with broadband and wide-angle RCS reduction has been presented in THz frequencies. The mechanism of low THz scattering was achieved by redirecting the reflective EM energies to all directions through the optimization of the arrangement of basic “00”, “01”, “10” and “11” digital elements. Hence this method can be applied as a coating of metallic targets with low RCS. The simulation and measured results show that the backward RCS of the metallic plate can be reduced by at least 10 dB in 0.7 THz–1.3 THz when coated with the ultrathin 2 bit coding metasurface. The numerical simulation shows that the broadband RCS reduction can also be achieved at the incident angle less than 60° for TE and TM polarizations. This work has opened a new route for achieving RCS reduction, which can be used for potential applications in the broadband THz radar stealth.

The perfect absorber is another approach to reduce RCS by absorbing all incident EM waves. Unike the absorber, the coding metasurface can cause diffusion of the reflected waves, which may redistribute the scattering energy, as it can not absorb all incident EM waves. This coding metasurface will not induce a change in temperature, and is much thinner than most traditional absorbing materials. However, there are some limitations in practical applications for the coding metasurface. First, the broadband reflection phase differentce characteristic was mostly designed for a flat object, therefore, it is challenging to reduce the wideband radar cross section for an arbitrarily shaped object. Second, an incident beam is mainly be scattered to two main beams, four main beam, etc by different coding sequences. The greatest disadvantage of this digital phase composition in continuous phase modulation is the control in arbitrary directions of the electromagnetic wave. Widespread applications necessitate further research of the coding metasurface for the effective manipulation of electromagnetic waves. This type of new metasurface will play an important role in radar, communication, and biomedical imaging.

## Methods

### Sample fabrication

The metasurface sample was fabricated using a standard photolithography process. First, a 40 μm-thick PI layer was fabricated by spinning-coated on a silicon substrate using liquid polyimide(viscosity, 3600 centipoise). Second, on top of this PI film consisting of 200 nm thick gold is patterned using photolithography and lift-off process. Third, the fabricated sample was peeled off from the silicon substrates using the HF solution for about 15 min. Finally, on the bottom of the polyimide film, a 200 nm-thick gold film was deposited by using an electron beam evaporator and the size of the entire sample is 2 inches.

## Additional Information

**How to cite this article**: Liang, L. *et al*. Broadband and wide-angle RCS reduction using a 2-bit coding ultrathin metasurface at terahertz frequencies. *Sci. Rep.* **6**, 39252; doi: 10.1038/srep39252 (2016).

**Publisher's note:** Springer Nature remains neutral with regard to jurisdictional claims in published maps and institutional affiliations.
